# An Overview of Healthcare Systems in Comoros: The Effects of Two Decades of Political Instability

**DOI:** 10.5334/aogh.3100

**Published:** 2021-08-18

**Authors:** Kassim Said Mohamed, Kassim Said Abasse, Muhammad Abbas, Dahiru Nasiru Sintali, Mirza Muhammad Faran Ashraf Baig, Andre Cote

**Affiliations:** 1 Department of Gastroenterology of Zhuhai Hospital, Sun Yat Sen University Guangzhou, China; 2 Institute for Chemical Carcinogenesis, Guangzhou Medical University, Guangzhou 511436, China; 3 Département de Management, Centre de Recherche en Gestion des Services de Sante, Faculté des sciences de l’administration (FSA), Université Laval (UL), Centre Hospitalière Universitaire (CHU) de Québec, Québec, G1V 0A6, Canada; 4 State Key Laboratory of Pharmaceutical Biotechnology, Nanjing University, Nanjing 210023, PR China; 5 Center For Research Development and Evaluation of Pharmaceutical Excipients and Generic Drugs, Department of Pharmaceutics, School of Pharmacy, China Pharmaceutical University, China; 6 State Key Laboratory of Analytical Chemistry for Life Sciences, School of Chemistry and Chemical Engineering, Nanjing University. Nanjing, 210023, China; 7 VITAM, Centre de recherche en santé durable, Université Laval, Quebec, QC, Canada

## Abstract

**Background:**

There is ongoing scientific evidence pointing out the adverse effects of conflict on population health and development. Union of Comoros has experienced nearly two decades of political instability and military rule. This comprehensive review was carried out to ask whether the health crisis in Comoros is attributable to the consequences of the chronic political instability.

**Methods:**

This study involved a series of semi-structured interviews with key informants complemented by a comprehensive literature search of electronic databases and grey literature. A literature search was performed using all identified keywords associated with health indicators in Comoros to identify potential eligible publications in both English and French from 1975 to July 2020.

**Results:**

The analysis demonstrated that political instability and lack of proper leadership from the Government undermine the establishment of health policies which contributed dramatically to the decline in health performance. Additionally, the resurgence and emergence of old and new diseases such as cholera, chikungunya, malaria, HIV/AIDS as indicators of inadequate health services were most likely during political turmoil. Data also showed an out-migration of the health workforce and an increased overseas medical treatment demand, which indicate less attractive working conditions and weak health systems in the country. Meanwhile, an increasing performance of health status indicators was observed after the comprehensive peace process of the 2000-Fomboni Declaration.

**Conclusions:**

The chronic political instability in Comoros has contributed to the health crisis facing the Union of Comoros. It has hampered the implementation of proper institutions, which might guarantee the socio-economic development and prosperity of the population. Further studies were needed to evaluate the health burden associated with the two decades of political instability and military rule.

## Introduction

Good health is the bedrock on which social progress is built. A nation of healthy people can do those things that make life worthwhile, and as the level of health increases, so does the potential for happiness [1]. However, political instability has dramatic impacts on societies, and it is often considered one of the main obstacles for economic, social, and political progress today [2,3,4,5,6]. Epidemiological studies indicate the devastating impact of conflict and/or political crisis on health [2,3,4,7,8,9,10,11,12,13,14]. For instance, Coghlan et al [11]. demonstrated that mortality rates in conflict areas within the Democratic Republic of Congo (DRC) were between two and three times higher than in the more stable areas.

Similarly, Berrang Ford, in her study, presents the characterization of the processes by which conflict has contributed to the occurrence of sleeping sickness outbreaks in Uganda, DRC, Sudan, and Angola [15]. Other studies investigating the effects of political instability on the health system have shown that in the crisis-affected region, for example, Goma in DRC, the HIV and TB control appeared not to be the focus of the health intervention despite the high risks of infection [14]. This could significantly increase the incidence of HIV infections and other sexually transmitted infections (STI) and jeopardize the country’s ability to cope with HIV/AIDS, likewise in Kenya during the 2007 post-election crisis [2]. Furthermore, the war in Syria [16,17], Lebanon [8,18], Bosnia Herzegovina [19], genocide in Rwanda [5,7,9,20,21], ethnic cleansing in Kosovo [22,23,24], and the invasion of Iraq led to the loss of lives [13,25,26], but deliberate destruction of health infrastructure, health workforce migration, reduction of financial resources, and decreased access to health service had a massive toll on the lives of survivors.

Meanwhile, the impact of political instability on health is not an easy task to identify since the exact causes of death and ill-health depend on a host of factors that include the size, intensity, and chronicity of the conflict, the state of existing infrastructure, and the extent of destruction of infrastructure, among others. Many reports of the relationship between political instability and health are based on small observational or survey-based studies. They often do not include correlations with levels of fighting or other relevant factors [4,27,28]. That is why this study aims to contribute to filling a literature gap in the interface of the above-mentioned factors.

## Political Context

The Comoros islands is located in the Indian Ocean, forming an Archipelago of four volcanic islands (Grande Comore, Anjouan, Mwali, and Mayotte), situated off the South-East coast of Africa, to the East of Mozambique and North-West of Madagascar. However, the sovereignty of Comoros only applies to the three islands of Grande Comore, Anjouan, Mwali, with Mayotte remaining a French Overseas Department as of March 31, 2011 [29,30,31]. This separation of Mayotte from the rest of the Federal Islamic Republic of Comoros (RFIC) (RFIC was the country’s official name at that time) was the starting point of political instability. Comoros has a long history of political instability and military rule since its incomplete independence from France on July 6, 1975. It has experienced over 21 coups or coup attempts, beginning just weeks after independence [30,31]. The Comorian political life has always been characterized by the presence of many political parties and political factions and a lack of consensus on how the country should be governed. The average duration of a government has not exceeded six months [30,31,32,33,34], worse than in most Sub-Saharan African (SSA) countries that experienced political instability after their independence (***Table 1***).

**Table 1:** Indicators of lack of state sovereignty in Africa.


COUNTRY	DURATION OF INTERNAL WARS AND STATE FAILURES	COUP EVENTS	RULERS ASSASSINATED

Burkina Faso		10	Sankara (1987)

Burundi	8 years and 5 months	1	Ndadaye (1993) Ntaryamira (1994)

Liberia	7 years and 1 month	10	Doe (1990)

Union of Comoros	25 years	21	Ali Soilih (1978) Abderemane (1989)

Mozambique	11 years and 9 months.		

Niger	6 months	1	Mainassara (1999)


In August 1997, the Comoros islands experienced a separatist uprising in the island of Anjouan during which time it (Anjouan), was not under the central administration [30,31,32,33,34]. The Government’s main efforts in these years of post-insurgency focused on the search for a solution to the political situation. Very complex negotiations were held in July and August 2000, which resulted in the signing of a peace declaration on 26 August 2000 with the Anjouan separatist party. The formalized Framework of Agreement called the Fomboni Declaration was signed on 17 February 2001, during which the international community’s support was instrumental [30,32]. This agreement reached by all the Comorian parties, with the aim of re-establishing the democratic political institutions and reunifying the country, gave rise to the setting up of a monitoring committee in which the international community took part and also led to the creation of a tripartite commission that comprised of government representative’s opposition parties and civil society organizations in the three islands. The 2000-Fomboni Declaration created a semblance of peace in the following years that led to a peaceful presidential election in May 2006 [30,31,32,34]. That ushered in the first democratically elected government since independence. This was a huge step in promoting national reconciliation, cohesion, and unity in Comoros.

## Socioeconomic Context

**Figure 1:**
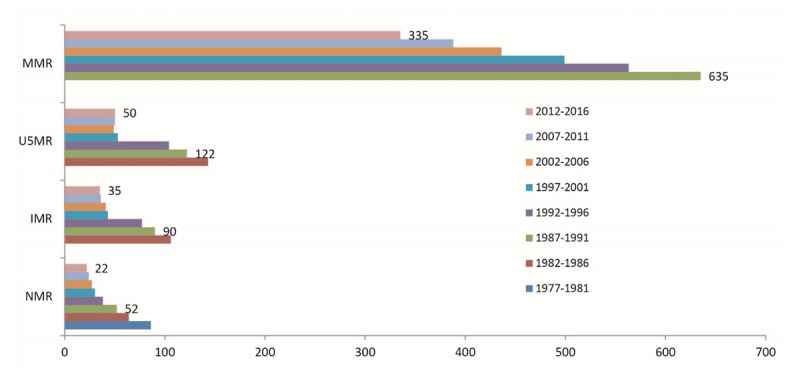
Trends of maternal mortality rate (MMR) and Child mortality rate in Comoros in 1970–2015.

The precarious political environment has hampered economic development by preventing good macroeconomic policies and undermining investor confidence. Comoros is one of the world’s poorest countries, with 54.7% of the population living below the poverty line in 2005 [30]. Moreover, Comoros’ return of political and institutional stability has allowed economic growth to resume, averaging 3% a year between 2011 and 2013. The outlook for 2019 was positive, supported by construction and private consumption sustained by money transfers from the Diaspora and by financial support and investments by foreign public and private partners [35,36,37]. Despite improved political conditions, economic activity has yet to recover from a long period of instability. For instance, the economic growth has done little to create jobs, which explains the high levels of unemployment estimated at 14.3% and youth unemployment at 44.5% [38]. Meanwhile, Human Development Index (HDI) has increased progress from 0.455 in 2005 to 0.538 in 2018, positioning the country at 156 out of 189 countries [37,39]. Moreover, some progress has been made as recent estimates of life expectancy have increased. The maternal and child mortality has decreased, and the fertility rate (as shown in ***Table 2*** and ***Figure 1***).

**Table 2:** Political, Demographic, Social and Economic Trends in Comoros from 1975–2015.


YEAR	GDP PER CAPITA 2011PPP$	FERTILITY RATE	LIFE EXPECTANCY AT BIRTH	THE % GDP	NET ODA RECEIVED (% OF GNI)

1975					36.11

1980	1,621	7.1	50.6		34.84

1985	1,700	5.3	53.8		41.59

1990	1,591	5.0	56.7		18.00

1995	1,473	4.8	58.7	4.5	17.86

2000	1,508	4.6	59.5	3.5	9.25

2005	1,492	4.4	60.1	4.3	6.03
	
2010	1,423		61.8	5.5	12.70
	
2011	1,424		62.2	5.9	8.86

2012	1,432	4.2	62.6	6.5	12.05

2013	1,447		62.9	5.8	13.26

2014	1,456		63.3	6.7	11.9


GDP per capita measured in purchasing power parity (PPP) equivalent dollars, reported as constant 2011 international dollar, based on estimates published by World Bank. TEH = Total Health Expenditure as % GDP. ODA = Official Development Assistance, GNI = Gross National Income.

## Materials and Methods

In assessing the impact of political instability on healthcare systems in Comoros. We administered a series of semi-structured interviews with key informants, comprising Comorian’s Health Administrators, Health Leaders of International and Non-Governmental Agencies in Comoros, from June to December 2019. A total of 20 key informants including five staff members from the Ministry of Health in Comoros, five staff members of World Health Organization (WHO), five staff members of United Nations Children’s Fund (UNICEF), two staff members of French Development Agency (AFD), two staff members of Chinese Embassy in Comoros and one staff member of Iranian Red Crescent Society, were interviewed in various health and health related issues, as well as their experience in promoting healthcare during and after political instability in Comoros. The semi-structured interview was designed to cover the six health system building blocks framework including service delivery [40,41], health workforce, information, medical products, vaccines & technologies, financing, and leadership/governance.

A literature search was carried out using PubMed/Medline, Google, Google Scholar, and other main data sources, using the word Comoros with a range of health-related terms including maternal health, child health, water sanitation, nutritional status of children, family planning, fertility, maternity care, immunization, infectious diseases, malaria, tuberculosis, and HIV/AIDS, to identify potential eligible publications in both English and French, from 1975 to July 2020. The detailed search strategy of the literature review is presented in the supplementary file (Appendix S1).

In addition, a comprehensive search of secondary information sources was also carried out through the WHO library database WHOLIS [42], the World Bank Documents and Reports repository [36], the United Nations Children’s Fund (UNICEF) [43], the United Nations Development Program (UNDP), the Demographic and Health Surveys (DHS) [44,45], and the African Development Bank (AfDB) [35]. For maintaining consistency and comparability in the sources of information, the most recent data (1990–2015) from the WHO Global Health Observatory Database were used to obtain information on maternal and child mortality rate, health expenditure, and exclusively the percentage of the population living on less than one dollar per day. We further classified these selected articles and/or reports accordingly to the time period of political history in Comoros.

Furthermore, the main purpose of this study is to highlight whether or not health outcomes in Comoros would have been better in the absence of a 20-year political instability. Thus, the study compares the prevailing conditions to an ideal state devoid of political instability. By so doing, the ramifications of political instability on the economy and health service delivery to the Comorian population is brought to the forefront. In particular, this study examines the resurgence of infectious diseases such as Malaria, Cholera, Chikungunya, and the high spread of TB/HIV infections and evaluates the trends in maternal and child health, as well as the health policies implemented before and after the 2000-Fomboni-Declaration, which has been arbitrary chosen in this study as the reference point of stability.

## Results

### Political instability and health care decline

Since its independence in 1975, Comoros, like many African countries, has inherited a health system characterized by massive disparities, with the more privileged urbanites having greater access to health care [46,47,48,49,50]. With the environmental and economic challenges and weak governance institutions and leadership since independence [30,32], the state of Comoros had experienced civil strife, as shown in the supplementary file (Appendix S2). It has also witnessed over 21 coups or attempted coups. In the midst of political turbulence and weak economic progress in which the average GDP growth was little over 0.6% in 1985 to 1996 together with an increase in population growth of 2.7 per annum, the Comoros government and the International Monetary Fund (IMF) agreed in 1990 to a structural adjustment program that lasted three years (1991 to 1993) [51]. The program proposed a plan whereby the government diversified its exports, reduced public expenditures, and privatized some of its parastatals. Furthermore, the plan called for the establishing user fees to access health services as in most SSA countries [52]. This led to under-utilization of healthcare services and drug shortages, as well as the resurgence in the use of traditional medicines (Appendix S3).

### Maternal and child health

***Figure 1*** shows the trends of maternal and child mortality rates from 1977 to 2015. Over the past 40 years, maternal (MMR) and child (under-five children [U5MR], infant [IMR], and neonatal [NMR]) mortality rates have fallen by more than half from 1987 to 2011. However, children continue to die from chronic malnutrition and diseases that can be prevented through immunization. Slow progress has been made on both maternal and child mortalities within this context, as shown in ***Figure 1*** and ***Table 3***. Furthermore, ***Table 3*** summarizes the progress made toward achieving the MDGs. Substantial progress has been made in the MDG6 with the control of malaria, which is as a result of partnership between the Comoros government and its partners such as the Global Fund Fight against HIV/AIDS, malaria and TB and also the Chinese cooperation [53].

**Table 3:** Trends in Estimates of Health Indicators in Comoros from 1990–2015.


	HEALTH INDICATORS	YEARS 1990–2015			CHANGE Δ(%)	ANNUAL RATE OF REDUCTION (AAR) (%)	MDGs TARGET2015	PROGRESS ON THE MDGS

		1990	2000	2015				

**MDG-1** Eradicate poverty and Hunger	% Stunted children	40^a^	47^c^	30^b^	25.0	1.15	10.30	No progress

	% Underweight children	21^a^	25^c^	15^b^	28.6	1.34	8.10	Insufficient

	% Population living below poverty line	54.7^g^	44.8^g^	45.6^g^	16.6	0.68	27.30	No progress

**MDG-2** Achieve Universal Primary Education	% Net enrolment ratio in primary Education	63.2^g^	69.4^g^	83^h^	–31.3	–1.10	100.00	Insufficient

	% Literacy rate among youth aged 15 to 24	59.1^g^	ND	90.0^g^	–52.30	–1.70	100.00	On track

**MDG-3** Promote Gender Equality and Empower Women	Ratio of girls to boys in primary education	0.85	0.86^g^	0.93^g^	–8.1	–0.36	1.00	On track

	Ratio of girls to boys in tertiary education	0.74	0.94	1.01	–36.50	–98.75	1.00	Achieved

	Proportion of seats held by women in national parliament	2	2	2	0.0	0.0	50%	No progress

**MDG-4** Reduce Child Mortality	Neonatal mortality rate (NMR) (deaths per 1,000 live births)	50^d^	38.2^a^	34^d^	32.0	1.53		Insufficient

	Infant mortality rate (IMR) (deaths per 1,000 live births)	88^d^	59^a^	55d	37.5	1.87	28.7	Insufficient

	Under five mortality rate (U5MR) (deaths per 1,000 live births)	125^d^	101^d^	74^d^	40.8	2.08	42.00	Insufficient

	% BCG vaccine coverage	90.8^a^	99.0^c^	84.6^b^	6.8	0.30	–––––	No progress

	% DPT3 vaccine coverage	68.6^a^	83.3^c^	71.2^b^	–3.8	–0.15	–––––	Insufficient

	% Measles vaccine coverage	87^g^	70^g^	85.3^g^	1.95	0.10	100.00	No progress

**MDG-5** Improve Maternal Health	Maternal mortality ratio (MMR) (deaths per 100,000 live births)	635^e^	499^e^	335^e^	47.2	2.6	157.50	Insufficient

	Total fertility rate	5.1^a^	4.4^c^	4.3^b^	15.7	0.84	–––––	Insufficient

	% Modern contraception	11^a^	ND	14^b^	–27.3	–0.97	>55	No progress

	% Antenatal care visit	85^a^	74^c^	92^b^	–8.2	–0.32	100.00	On track

	% Skilled attendant at delivery	52^a^	62^c^	82^b^	–57.7	–1.84	100.00	Insufficient

	% Delivery births at health facility	42.5^a^	ND	76.1^b^	–79.1	–2.36	–––––	Progress

	% Delivery births at home	57.0^a^	ND	21.8^b^	61.8	–3.78	–––––	Progress

	% Family Planning needs unsatisfied	50^a^	ND	31.6^b^	36.8	1.82	15	Insufficient

**MDG-6** Combat HIV/AIDS Malaria and Other Diseases	Malaria prevalence	33	34.5	9	72.73	5.06	16.5	Achieved

	Proportion of children under-five sleeping under ITNs	ND	9	61	–577.78	–13.61	100.00	On track

	Tuberculosis prevalence	32	15	ND	53.13	7.30	16	Achieved

	Proportion of tuberculosis cases detected and cured	77	94.5	95	–23.38	–0.84	100.00	On track

	HIV prevalence among population aged 15-24 years	ND	0.023^g^	0.05^g^	–117.40	–5.31	0.025	Insufficient

	Proportion of the population aged 15-24 years with comprehensive correct knowledge of HIV/AIDS	ND	1.6	67.8	–4,137	–28.40	100.00	On track

**MDG-7** Ensure environment sustainability	% Population using improved drinking water source	87^f^	92^f^	70.6^b^	18.85	0.83	100.00	No progress

	% Population using improved sanitation	18^f^	28^f^	ND	–55.56	–4.52	27.00	Achieved

**MDG-8** Develop a Global Partnership for Development	Proportion of total bilateral and ODA allocated to basic social services (basic education, primary health care, nutrition, safe water and sanitation)	45^g^	47^g^	55^g^	–22.23	–0.81	80	Progress

	% Population who are cellular or mobile subscribers	ND	0.34^f^	32.3^f^	–9,400	–35.50	–––––	Progress


*Note*: a = the figures are for 1996 DHS. b = the figures are for 2012DHS. c = the figures are for 2000MICS. d = Estimates by the UN inter-agency Group for Child Mortality Estimation [47]. e = Estimates by the UN inter-agency Group for Maternal Mortality Estimation [41]. f = WHO -Comoros factsheets of health statistic 2014 [43]. g = Comoros –MDGs Rapport 2013 [49]. h = the World Bank Data [56].

### Health financing

**Figure 2:**
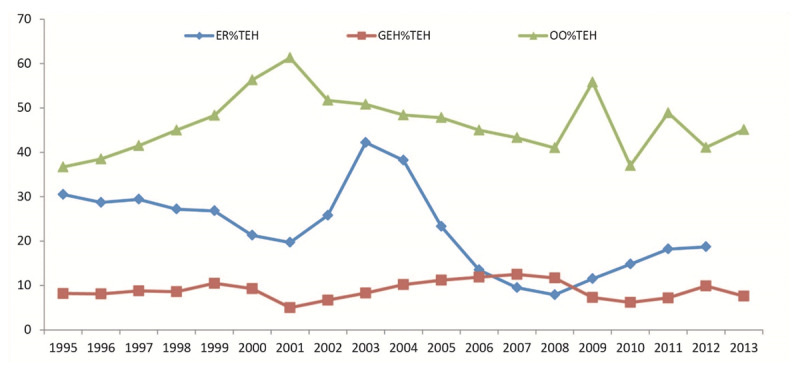
Trends of financing related to the contributing of out-of-pocket (OO%TEH), external resource (ER%TEH) and Government Expenditure on health (GEH%TEH) as %of Total Expenditure on Health in Comoros (1995–2014).

National Health Account (NHA) data shown in ***Figure 2*** illustrates the general government expenditure on health on health (GE%TEH) from 1995 to 2013 in Comoros 5% to 10%. Per capita, GE%TEH ranges from a mere $30 to $50. However, the out-of-pocket payment as a percentage of total expenditure on health (OO%TEH) was over 50%. Meanwhile, foreign aid as a share of total health expenditure (ER%TEH) ranged from 10% to 30%, which was observed when there was e political stability in Comoros. From 1997 to 2000, the out-of-pocket payment contributed to over 60% of the total health expenditure. At the same time, the ER%TEH and GEH%TEH have been remarkably declined due to the secessionist crisis in Anjouan island (as shown in ***Figure 2***).

### Resurgence and emergence of old and new diseases

The most common diseases in Comoros are malaria, diarrhea, intestinal parasites, and respiratory infections (ARI). These conditions have caused significant morbidity and high mortality, especially among children under five and pregnant women. HIV/AIDS and other infectious diseases are also a major concern despite their relatively low prevalence, as shown in Appendix S3.

### Malaria disease

Malaria has been the leading cause of morbidity and mortality in Comoros since 1923. In 1954, multiple malaria prevention and control interventions were started but were suddenly abandoned after independence. However, in early 1988, with renewed commitment to tackle the malaria problem, the Ministry of Health (MoH) of Comoros developed some malaria control policies, which led to the establishment of the National Malaria Control Programme (PNLP) charged with the responsibility to spearhead and coordinate all malaria prevention and control interventions. According to PNLP, the malaria incidence was estimated at 26.2 per 1,000 populations in 1996, and it was the main cause of hospitalization in health facilities. Within that same year, malaria accounted for 45% and 8% of hospital consultations for children under five and pregnant women respectively [45,49,51,54]. However, substantial progress has been made in recent years, leading to the decline of malaria incidence to less than 1 case per 1,000 people, according to Kassim et al. (2016) (Appendix S3) [55].

### Sexually transmitted infections (STI) and HIV/AIDS

According to the Comoros islands, the HIV prevalence rate is estimated at less than 0.025% in the 2003 Seroprevalence survey. While this rate may be considered low, there is a tendency to increase if appropriate measures are not taken. The high prevalence of at-risk factors justifies the concern over the potential spread of HIV. These risk factors include the low rate of condom use estimated at 27%, the low detection rate of 8%, and the relatively high prevalence of STIs (4.6%) [56]. Data from hospital records and those obtained from the national infectious diseases control program put the number of HIV-infected people between 1988 and 2008 in Comoros at one hundred and six (106) [56]. Over half of this group were females (66.1%) and between 19–34 years (55.7%). In addition, 40 deaths of these cases were reported, with 17 and 49 still alive and lost to follow up respectively.

On the other hand, Tuberculosis remains quite widespread and poses a potential risk with the advent of the AIDS epidemic. The annual risk of infestation is 60 per 10,000 inhabitants, with a detection rate of 24.5 per 100,000 inhabitants. The mortality rate is of the order of 0.5 per 100,000, while the lethality rate is 2.15 per 100,000 inhabitants. In 2004, 102 cases were recorded at the national level, and 72% were from Grande Comore. The tuberculosis prevalence in 2008 was estimated at 55 per 100,000 inhabitants (Appendix S3).

### Cholera outbreak in Comoros from 1975–2007

The first cholera outbreak in Comoros was reported in 1975 and accounted for 2675 cases and 238 deaths (case fatality rate of 8.9%) [57,58]. In 1998, the second-largest outbreak occurred, in which 7300 cases and 68 deaths were reported. From 1999 to 2004, cholera cases were reported each year, with a case fatality rate ranging from 0 to 3.56%. Also, between 2001 and 2002, the outbreak accounted for 1644 cases with 47 deaths (CFR 2.9%) in all three islands [58,59]. The epidemic started in Grande Comore and spread to others. The country’s attack (infection) rate was 0.3% and was higher in Grande Comore (0.4%) than in the two other islands. In addition, the outbreak in 2007 started on 25 February in Grande Comore (Moroni) and reached a peak of more than 400 weekly cases by August. Towards the end of the year, 1531 cases and 29 deaths were reported, of which 96% of the cases were from Grande Comore and 4% from Moheli Island, but Anjouan Island was unaffected. The overall CFR in Comoros was estimated at 1.9% [57,58,59,60].

### Chikungunya outbreak in Comoros, 2005

Chikungunya is a viral disease (genus Alphavirus) that is transmitted to humans by infected mosquitoes. This arthropod-borne viral disease was first described in Tanzania in 1952 [61]. Since 1952, the Chikungunya virus (CHIKV) has caused infection outbreaks in several SSA countries and Asia [61]. In 2004, outbreaks of CHIKV infection were recorded in coastal Kenya (Mombasa and Lamu Island), and during 2005, an outbreak occurred in the Comoros [62,63,64]. From January to April 2005, an epidemic of CHIKV illness occurred in Grande Comore, in which over 1,100 cases were reported [62,63,64].

### Health workforce migration

In SSA countries, the shortage of health workforce is one of the key determinants of a weak health system. ***Table 4*** presents the trend in the workforce of the health sector in Comoros from 2002 to 2012. While the Comoros islands, since its independence continues to face challenges on shortages and imbalances in the geographical distribution of health personnel that are not highly skilled, low productivity and poor motivation have been the hallmarks of the health workforce in Comoros and has undisputedly undermined the performance of the health care systems. A solid health workforce policy is needed to tackle the above-mentioned challenges and strengthen the health care system. In 2006, WHO identified Comoros as one of 57 countries with critical deficit in the supply of skilled health workers [43]. For instance, Michael Clemens et al [65]. estimated in their study that 32% of Comoros-born physicians and 23% of Comoros-born nurses are working in one of nine developed countries [65]. This same is observed for other SSA countries [65]. This leads to the urgent need for strengthening health workforce policies to improve the performance of health care systems and enable them to better provide accessible, efficient, safe, and effective services in SSA countries, particularly in Comoros.

**Table 4:** Evolution of Health Workforce in Comoros 2002–2012.


NUMBER (DENSITY PER 1000 POPULATIONS)	2002	2012

Physicians	115 (0.146)	197 (0.268)

Nurses and midwives	588 (0.744)	576 (0.784)

Dentists and technicians	29 (0.037)	27 (0.037)

Pharmacists and technicians	41 (0.052)	29 (0.04)

Environmental and public health workers	17 (0.022)	ND

Laboratory technicians	63 (0.080)	82 (0.111)

Other health workers	9 (0.011)	725 (0.986)

Community health workers	41 (0.052)	ND

Health management and support	272 (0.344)	122 (0.166)

Sum Total	1175 (1.487)	1758 (2.391)


### Migration of Comorian population to access health care

With the above-mentioned challenges and high user fees in the health facilities in Comoros, most of the Comorian population migrated to neighboring countries, where they could have access to quality and affordable health care. A recent study conducted in Mayotte found that the proportion of Comorians who had emigrated for health reasons was 8.8% in 2005 [66]. Another study estimated 115 patients per year between 2004 and 2007. In addition, the French national institute of health and medical research (INSERM) estimated around 11% of the proportion of the Comorian population migrated to Mayotte for medical treatments in 2008 [67,68]. However, since the “Visa Balladur” implementation in 2005, most Comorians migrated to Madagascar, Tanzania, and Mauritius [66,67,68,69]. Although no recent studies are available, the most recent study gave an estimated number at about 10 times more of Comorians who travel to Madagascar to seek medical care [70].

### Political stability and health care

After the Fomboni Declaration, the Comoros government and the International Community set up a strategic plan to strengthen fragile peace and promote good governance. As mentioned above, several agreements between the Comoros government and its partners have allowed the government to benefit from the PRGF and HIPC program. Political stability has created opportunities for socio-economic development, as shown in ***Table 2*** and ***Figure 1***. ODA has increased, allowing the government to increase its contribution to health expenditure, after its suspension during the political and institutional instability (1997–2007). Currently, ODA is the most significant contributor to total health expenditure (THE), around 37% in 2013, mainly benefiting vertical programs. Additionally, the Comoros Government, with the Chinese government’s financial support in 2007, set up the Fast Elimination of Malaria by Source Eradication (FEMSE) project, which led to the successful eradication of malaria in Comoros. According to Kassim et al. [55], Comoros has made significant progress on malaria control in the past few years, currently putting the country in the pre-elimination phase. In response to the socio-economic challenges in Anjouan and the vast disparities among the population, the Chinese government and other international partners, including the Iran Red Cross and Fatima Foundation, built hospitals and opened primary health care centers to strengthen the health system. In 2006, UNICEF launched several initiatives to promote the maternal and child health and education within islands [43]. All these initiatives have dramatically enhanced the Comoros government’s efforts towards achieving the MDGs [71], as shown in ***Figure 1*** and ***Table 3***. Maternal and child mortality has declined to more than half due to the substantial decrease in malaria deaths and the increase in skilled birth attendants in 2012 [72]. Also, various initiatives aimed at strengthening the primary health care initiated by the Government with its partners have considerably improved maternal and child health after the Fomboni Declaration (See ***Figure 1***), resulting in great improvement in achieving MDGs (***Table 3***). However, a lot has to be done to reduce poverty and hunger, which remain vital challenges facing the population in Comoros. Most recently, Comoros was ranked as the third hungriest nation in the world [71]. As a response to such difficulties, the Comorian Government developed a National Strategic to reduce poverty and accelerate economic growth to reduce the socioeconomic disparities among the population. However, its implementation is still a far-fetched idea.

## Discussion

To the best of our knowledge, this study is the first of its kind to be carried out in the Comoros islands that describes and analyzes political instability and its impact on the health and the population. It was argued that the political instability had hampered economic development by preventing the implementation of good socio-economic policies geared toward tackling the country’s challenges. Furthermore, the lack of proper planning on the part of the Government has remarkably contributed to the decline of appropriate and affordable health services. This study has reviewed the health status and its trends since the independence, analyzing the progress made, challenges faced before and after the peace agreement, and highlighted areas that need improvement.

This led the government to implement the structural adjustment program recommended by the IMF in 1992 toward reducing the public expenditure and privatizing its state-owned companies. However, this intervention led to a drastic reduction or a complete halt in financing key public health programs such as malaria control and accessible healthcare services, including drugs. A weak public health system was thus created, incapable of quickly responding to health threats such as the Cholera and the Chikungunya outbreaks in 1975 and 2005 respectively, and the spread of HIV/AIDS, as reviewed above [57,58,59,73,74]. Similar studies conducted in other SSA countries arrive at the same conclusion [2,10,14,15,60,61,64]. In addition, as shown in ***Figure 2***, the Government health expenditure and External resource for health as % of THE dramatically decline during the political turbulence, attaining its minimal point of 4.98% and 19.51% respectively in 2001. In such a situation, the out-of-pocket health expenditure as % of private health expenditure was 100%, which harms household consumption and ultimately an impoverishing effect on households and a decline in the use of health service [71,75].

Moreover, the Government and its international partners have taken considerable engagement by financing the health system, which has reduced the out-of-pocket payment by 40% since 2010. In terms of the health workforce, Comoros, like many African countries, faces a huge migration challenge of healthcare personnel to developed countries [67,68]. The latter situation has fragilized the quality of health services which could also be explained by the high overseas medical treatment experienced by the population [66,70]. It was clear that the Fomboni-Declaration has created a semblance of peace [30,32], which led to a peaceful presidential election in May 2006 that promoted national reconciliation and cohesion. In this unity of Comoros, various investors and partners have got confidence.

After 2000 Fomboni-Declaration, the political stability has led to investor confidence and implementation of the proper macroeconomic policies geared toward improving the country’s GDP growth. In 2011, Comoros experienced a 3% growth in GDP which impacted the health care systems immensely [35,36,37]. Several achievements in the health sector have been made within this short period (***Table 3***), such as a decline in maternal and child mortality, an increase in net enrolment, an achievement on malaria HIV/AIDS, and TB control. The health care system has improved substantially though efforts are needed to tackle the remaining challenges, MDG1.

### Limitation of this study

Despite the relevant source of information on socioeconomic and health indicators provided by demographic health surveys, millennium development reports, several survey studies, and international organizations such as WHO, UNICEF, World Bank databases of Comoros over time, this study remain a retrospective analysis based on secondary data with all the limitations of using such types of data sources. Although the study pointed out that only the political instability was the cause of the health outcomes, those factors contributed to several biases. Further research on governance and health policy in Comoros would allow a more thorough analysis of the causes of progress or lack of progress observed for the past four last decades. It will reveal in more detail the causes of health outcomes and recommended the policy responses that Comoros may have adopted to address the demand and supply side factors that were not addressed in this study, such as health policy, social and cultural factors, and lack of education, infrastructure and information.

## Conclusion

This overview attempts to establish the link between the Comorian population’s political instability and health outcomes by reviewing the variable opportunities lost during the political and institutional instability. Thus, the study compares the overall health status to population health status in the political instability. By doing so, the study supports the hypothesis that political instability adversely affects health performance by undermining the planning and/or implementing the appropriate health policies that could assure health equity and improve the well-being of the people. Our analysis has shown the adverse effects of political instability in various health indicators, including leadership and governance, health financing, human resources, and health services quality. Substantial progress has been made after the Fomboni Declaration, such as reduction increase of health expenditure and ODA, reduction of maternal and child mortality, control and elimination of infectious diseases including malaria, tuberculosis, and increase of human development index.

## Data accessibility Statement

All datasets generated for this study are included in the article/supplementary material.

## Additional Files

The additional files for this article can be found as follows:

10.5334/aogh.3100.s1 Figure S1. History of political instability in Comoros.

10.5334/aogh.3100.s2 Appendix S2. Search Strategy.

10.5334/aogh.3100.s3 Appendix S3. List of selected studies with their health crises and implication in Comoros.
