# Supplementary material for: An Overview of Healthcare Systems in Comoros: The Effects of Two Decades of Political Instability
Source: Ann Glob Health. 2021 Aug 18;87(1):84. doi: 10.5334/aogh.3100 (PMC8378088; doi:10.5334/aogh.3100)
Supplement: Figure S1. — History of political instability in Comoros. [file agh-87-1-3100-s1.pdf]

Supplementary file

Figure S1: History of political instability in Comoros

- ✓ **1973-**Agreement between France and Comoros to become independent in 1978
- ✓ **July 6, 1975-**Comorian parliament passed a resolution declaring independence; however the deputies of Mayotte abstained.
- ✓ **September 5, 1975-**Ahmed Abdallah become the first President of Comoros
- ✓ **August 3, 1975-**Bob Denard French Mercenary removed President Ahmed Abdallah and replaced him by Prince Said Mohamed Jaffar.
- ✓ **January 1976,** Jaffar was ousted in favor of Minister of Defense Ali Soilih
- ✓ **February 1976**, the population of Mayotte voted against independence from France
- ✓ **In 1978,** B. Denard returned and overthrew A.Soilih by force and reinstated M.Abdallah
- ✓ **In 1989,** B.Denard killed President Abdallah and Said Mohamed Djohar became the president
- ✓ **In 1995,** B.Denard returned and attempted another coup removed President Djohar
- ✓ **From 1996-1997**, Mohamed Taki Abdulkarim became president and the secessionist conflict started in Anjouan and Moheli in 1997.
- ✓ **In 1998-**President Taki died and he was succeeded by interim President Tadjine ben said Massoundi
- ✓ **April 1999**, Colonel Azali Assoumani takeover the Comoros' eighteenth coup d etat since independence 1975. Azali negotiated the 2000 Fomboni Agreements for power-sharing in which the federal presidency rotates among the three islands and each island maintains its own local government
- ✓ **December 26, 2001**, 75% of the population of the Comoros voted in favor of a new constitution to reunite the three islands and end the secessionist crisis. The basis of this settlement was codified in the Fomboni Agreement.
- ✓ **May 26, 2006**, Mohamed Abdallah Sambi became the first peaceful and transparent president under the new constitution. Since then fragility stability prevail in Comoros.
