# Supplementary material for: An Overview of Healthcare Systems in Comoros: The Effects of Two Decades of Political Instability
Source: Ann Glob Health. 2021 Aug 18;87(1):84. doi: 10.5334/aogh.3100 (PMC8378088; doi:10.5334/aogh.3100)
Supplement: Appendix S2. — Search Strategy. [file agh-87-1-3100-s2.pdf]

Appendix S1: Search Strategy

Database	Keywords
**PubMed**	((“Comoros” [Title/Abstract] AND “Healthcare Delivery System” [Title/Abstract] OR “Leadership” [Title/Abstract] OR “Socioeconomic” [Title/Abstract] OR “Governance” [Title/Abstract] OR “Political Instability” [Title/Abstract] OR “Health disparities” [Title/Abstract] OR “WHO building blocks” [Title/Abstract] AND “Healthcare Financing” [Title/Abstract] OR “Health Workforce” [Title/Abstract] OR “Medical technologies” [Title/Abstract] OR “Medical products” OR “Research” OR “Quality of service delivery” OR “Health Indicators” OR “life expectancy” [Title/Abstract] OR “Health Outcomes” [Title/Abstract] “Maternal and child mortality” [Title/Abstract] OR “Maternal and child health” [Title/Abstract] OR “Infectious diseases” OR “Chronic diseases” [Title/Abstract] OR “Water sanitation” [Title/Abstract] OR “Access to health care” [Title/Abstract] OR “accessibility of health services” [Title/Abstract] OR “assessment of healthcare needs” [Title/Abstract] OR “assessment healthcare quality” [Title/Abstract] OR “assessments healthcare” [Title/Abstract] OR “administration health service” [Title/Abstract] OR “health service” [Title/Abstract] OR “health policies” [Title/Abstract] OR “national health policy” [Title/Abstract] OR “national health policies” [Title/Abstract] OR “accountable care organizations” [Title/Abstract] OR “health expenditure” [Title/Abstract] OR “health expenditures” [Title/Abstract] OR “health infant service” [Title/Abstract] OR “health information technologies” [Title/Abstract] OR “catastrophic health insurance” [Title/Abstract] OR “comprehensive health insurances” [Title/Abstract] [MeSH Terms]).
	((“Comoros” AND “Healthcare system” OR “Governance” OR “Leadership” OR “Socioeconomic” AND “Healthcare system” OR “Health disparities” OR “Political Instability” OR “WHO building

**The Cochrane Library**	blocks" OR "Healthcare Financing" OR "Health Workforce" OR "Health coverage" OR "Medical technologies" OR "Medical products" OR "Research" OR "Information" OR "Health Indicator" OR "Life expectancy" OR "Health Outcomes" OR "Infectious diseases" OR "Chronic diseases" OR "Maternal and child health" OR "maternal and child mortality" OR "Access to health care" OR "accessibility of health services" OR "assessment of healthcare needs" OR "assessment healthcare quality" OR "assessments healthcare" OR "administration health service" OR "health service" OR "health policies" OR "health policies" OR "national health policy" OR "national health policies" OR "accountable care organizations" OR "health expenditure" OR "health expenditures" OR "health infant service" OR "health information technologies" OR "catastrophic health insurance" OR "comprehensive health insurances").
**Embase**	'Comoros' OR 'Politics' OR 'Governance' OR 'Leadership' OR 'Socioeconomic' AND 'Healthcare system' OR 'WHO building blocks' AND 'Healthcare Financing' OR 'Health Workforce' OR 'Health coverage' OR 'Medical technologies' OR 'Medical products' OR 'Research' OR 'Information' OR 'Health Indicator' OR 'Political Instability' OR 'Life expectancy' OR 'Health Outcomes' OR 'Infectious diseases' OR 'Chronic diseases' OR 'Maternal and child health' OR 'maternal and child mortality' OR 'Access to health care' OR 'accessibility of health services' OR 'assessment of healthcare needs' OR 'assessment healthcare quality' OR 'assessments healthcare' OR 'administration health service' OR 'health service' OR 'health policies' OR 'health policies' OR 'national health policy' OR 'national health policies' OR 'accountable care organizations' OR 'health expenditure' OR 'health expenditures' OR 'health infant service' OR 'health information technologies' OR 'catastrophic health insurance' OR 'comprehensive health insurances'.

**Web of Science**	(Comoros) OR (Governance) OR (Leadership) OR (Socioeconomic) AND (Healthcare system) AND (WHO building blocks) OR (Healthcare Financing) OR (Health Workforce) OR (Health coverage) OR (Medical technologies) OR (Medical products) OR (Research) OR (Information) OR (Health Indicator) OR (Political Instability) OR (Life expectancy) OR (Health Outcomes) OR (Infectious diseases) OR (Chronic diseases) OR (Maternal and child health) OR (maternal and child mortality) OR (Access to health care) OR (accessibility of health services) OR (assessment of healthcare needs) OR (assessment healthcare quality) OR (assessments healthcare) OR (administration health service) OR (health service) OR (health policies) OR (health policies) OR national health policy) OR (national health policies) OR (accountable care organizations) OR (health expenditure) OR (health expenditures) OR (health infant service) OR (health information technologies) OR (catastrophic health insurance) OR (comprehensive health insurances).
**Google Scholars**	Comoros AND Healthcare system OR Politics OR Governance OR Leadership OR Socioeconomic AND Healthcare system AND WHO building blocks OR Healthcare Financing OR Health Workforce OR Health coverage OR Medical technologies OR Medical products OR Research OR Information OR Health Indicator OR Political instability OR Life expectancy OR Health Outcomes OR Infectious diseases OR Chronic diseases OR Maternal and child health OR maternal and child mortality OR Access to health care OR accessibility of health services OR assessment of healthcare needs OR assessment healthcare quality OR assessments healthcare OR administration health service OR health service OR health policies OR health policies OR national health policy OR national health policies OR accountable care organizations OR health expenditure OR health expenditures OR health infant service OR health information technologies OR

	catastrophic health insurance OR comprehensive health insurances.
