# Supplementary material for: An Overview of Healthcare Systems in Comoros: The Effects of Two Decades of Political Instability
Source: Ann Glob Health. 2021 Aug 18;87(1):84. doi: 10.5334/aogh.3100 (PMC8378088; doi:10.5334/aogh.3100)
Supplement: Appendix S3. — List of selected studies with their health crises and implication in Comoros. [file agh-87-1-3100-s3.pdf]

Appendix S3: List of selected studies with their health crises and implication in Comoros

First author, year	Health crises	Aims of study	Study design	Health outcomes	Challenges	Limitation of the study
Said Abasse Kassim et al.,2017 [1]	Health inequalities in across islands	Assessment of healthcare services in urban and rural areas, as well as across islands of Comoros.	A cross-sectional surveys	• Healthcare service inequality across islands<br>• Maternal and Child health disparities<br>• Improving health of rural areas by providing cash to vulnerable households.	• Poor access to healthcare services<br>• Impact of delivering small cash to improve health disparities of rural areas was difficult to evaluate.	• Response to health policies was not addressed in this study.
Dario Donno et al. 2018[2]	Malnutrition and food insecurity	Evaluating the phytochemical composition of jams and jellies prepared from seven fruits of Comoros	Extraction and analytical methods (i.e. Spectrophotometric and chromatographic methods)	• Phytochemical and nutraceutical properties of tropical fruits.<br>• Jam and jellies prepared from tropical fruit are rich source of polyphenolic constituents.	• Identification of chemical markers and fingerprints of jam and jellies	• The analytical technique restricts its applicability.
Abayomi Samuel Oyekale et al., 2018[3]	Child mortality	Role of maternal education and fertility on child survival in Comoros	Survey	• Child mortality in urban area is 30% lower than rural area. So, a child health policy is needed to educate about child health.<br>• Malaria ranked second major cause of premature death among Comorians between 1990 and 2010 and result of this study indicated that mosquito nets increased survival among infant children.	• Lack of basic social services in rural areas<br>• Low education level of women	• Despite the fact that authors did not highlight the limitation of study. But it is clear the study design presents several limitations.
Blessing J Akombi et al. 2018[4]	Malnutrition status	Prevalence of Malnutrition in	Meta-analysis	• Highest burden of malnutrition in East Africa and West Africa compared to WHO target.<br>• This study recommended an urgency of strategic	• Poverty is the main reason of undernutrition in vulnerable regions.	• DHS data (2006–2016) of few Sub-Saharan Africa countries was not available.

				policy to improve child nutrition in the vulnerable countries.		
A Ouledi et al., 2012[5]	Presenting the status of healthcare systems in Comoros	Overview of health history and challenges in Comoros	Overview of the healthcare systems	• This study demonstrated an improvement of health and social progress.<br>• Progress has been made recorded in vector borne diseases, such as malaria and arboviruses, and to cholera.<br>• The prevalence of AIDS is 0.025%.	• Epidemiological features dominated by communicable diseases (malaria, diarrheal diseases, respiratory diseases, and gastrointestinal parasites).<br>• Major upsurge in the prevalence of non-communicable diseases (cyclones, floods, volcanoes and tsunamis	• This study presented some limitations due to its design (An overview of secondary data analysis)
Anne-Sophie Ruget et al.2019[6]	Peste des petits ruminants virus outbreak	Geographic Information System (GIS)-based Multi Criteria Evaluation (MCE) used to identify areas at risk of PPR occurrence and spread in Comoros and other four Eastern Africa countries.	Spatial Multicriteria Evaluation	• Mapping helps in disease surveillance and control measures, which can eradicate the PPR goal of OIE and FAO by 2030.<br>• The results suggested that the GIM-MCS have a reliable predictive accuracy and could be used for risk-based surveillance and control purposes.	• Data quality	• Computational limitations.
Nimer Ortuno-Gutierrez et al.,2019[7]	Leprosy incidence	This trail will asses the effectiveness of different approaches of Post-exposure prophylaxis (PEP) on the Comoros and Madagascar.	A cluster-randomized trial	• This study has demonstrated the protocols of PEOPLE a cluster randomized trial.<br>• Digital tools for mapping allow geospatial patterns in leprosy transmission.	• Participant recruitment.	• The reliable incidence data for recent years are not available.
Nasserdine Papa Mze et al., 2015[8]	Diagnosis of suspected malaria cases.	Fast and accurate detection of plasmodium in the blood	Rapid diagnostic tests (RDTs)	• RDTs was validated as a source of DNA for determining drug	• drug resistance monitoring	• Limitation of the study was not mentioned.

				resistance mutations.<br>• A policy is needed to evaluate yearly SP drug resistance, which can assist to assess SP efficacy in future.		
Luis de Almeida et al. 2019[9]	food contaminant	Determine the levels of aflatoxin in in blood samples of women and children.	Grain and blood samples analysis and spatial analysis	• Afatoxin contaminated grain is a causative factor of malnutrition.<br>• This study revealed that there is no correlation found between aflatoxin albumin levels and stunting.	• Drying grain	• The urine testing for metabolites of afatoxin has exposure limitation.
Saindou Ben Ali Mbaé et al.,2016[10]	Food poisoning	Find the Source and control of food poisoning outbreak	a case-control study	• sea turtle meat and Eretmochelys imbricata were indicated the source of the outbreak.<br>• This study also reported that cooking does not destroy the toxin.	• Number of cases were more than controls.	• Cases were overestimated in the selection process.
B-A Gaüzère et al. 2013[11]	Overiew of history of human epidemic and endemic diseases in the southwest Indian Ocean	This study aims to review the history epidemic and endemic diseases such as Smallpox, HIV/AIDS, Dengue, Malaria, Leprosy, and Cholera.	Descriptive study	• Malaria disease remains prominent in Madagascar and Comoros, since 1841.<br>• Leprosy has been known in La Réunion since 1726 and is still very present in Mayotte, Anjouan, and Madagascar.<br>• Dengue, chikungunya, and Rift Valley fever are also present.	• Various challenges have been pointed out concerning the control and prevention of emerging infectious diseases.<br>• Low health expenditure<br>• Lack of health infrastructure	• Despite the fact did not present the limitation of study, but it is clear that some limitations might be pointed out due specially for its design.
Charlotte Avanzi et al., 2020[12]	Spread of infectious disease i.e. leprosy	Identify the relationship of human migration and infectious diseases.	Genotype screening and DNA extraction	• Low strain diversity in Comoros and Madagascar compared to other islands<br>• specific 1D-Malagasy marker on the islands can retrace the exact origin of the 1D-Malagasy	• The Malagasy genomic information was inadequate.	• Ruled out the leprosy in the Austronesian settlers.

				genotype.		
Rehana A Salam et al.,2015[13]	Maternal health indicator	Analyze the available literature on nutrition health and improving maternal nutrition	Meta-analysis	• multiple nutrition programs are running in the ESAR with different scale.<br>• Countries with highest burden of maternal undernutrition needs to be prioritized for improving maternal health.	• Insufficient maternal health and nutritional indicators due to the scare nutritSion specific program.	• Poor health system.
Ali H Mokdad et al., 2014[14]	Disease burden	Assessing the disease and injuries burden in 22 Arab countries	Survey	• Disease burden has majorly affected the human and financial resources of Arab countries.<br>• There is an urgent health policy is needed to reduce the disease burden in Arab countries.	• Weak health database	• Lack of data
Yasmina Dada,et al., 2007[15]	Communicable disaeses	Assessing the number of HIV and STI cases in Comoros	cross-sectional surveys	• HIV cases are rare in the Comoros compared to the world.<br>• HIV control program focus specially on sex workers.	• It was difficult to estimate the exact number SWs in Comoros.	• lack of testing facilities.
Frédéric Pagès et al., 2018[16]	Imported malaria	Assessment of clinical and epidemiological features of malarial patients and their prevention practices	in vitro testing	• Low risk of reoccurrence of malaria in Reunion.<br>• Regular travelers to other islands specially to Madagascar, are high-risk group of contracting malaria.	• Difficulty in assessing data	• Traveler information was limited
Ali Mohamed Elyamine etal., 2018[17]	Promoting the cadmium decontamination in soil	Evaluating the effect of cadmium in soil and its uptake by plants	Analytical tests	• The findings suggest that the earthworms, rice straw and plants could interactively increase the decontamination of Cadmium contamination in the soil.	• Limited literature was available on this topic.	• Limitation of the study was not mentioned.

				• This study provides a better insight of soil biological interaction effects on metal remediation technology.		
Samson Gebremedhin et al., 2015[18]	Obesity in preschool children	Assessment of BMI in preschool children in SSA region	Cross-sectional	• Obesity is the major health burden in the SSA region.<br>• There was no significant association was observed with GDP, residence (urban–rural) and household income.	• No birth weight data was available.	• All countries in SSA were not included in this study.
Gopal K. Singh et al.,2012[19]	Cervical Cancer	Inequalities in incidence and prevalence of cervical cancer	Log-linear regression models	• The incidence of cervical cancer can be reduced by providing a better health services and women social status.<br>• This study also revealed that increase health can significantly decline the incidence and mortality risks of cervical cancer.	• Detection of cancer at an early stage	• Limitation of the study was not mentioned
Zainab A. AlQarni et al.,2016[20]	Health promotion on Facebook	Assesses the knowledge gap for DM	A mixed-methods quantitative and qualitative method	• This study revealed that diabetes patients shares more information about their health status on Facebook.<br>• This study highlights the pivotal role of social media in improving public health.	• Professional and ethical challenges	• Limitation of the study was not mentioned
Guillaume Camuset, et al.,2016[21]	Leprosy	Annually incidence rate of leprosy and patients characteristics	An observational study	• Leprosy is no longer a major health burden but reoccurrence threat will always remain.<br>• Still, there is need to detect active cases and treat them rapidly.	• Preventing resurgence is major challenge	• Undeclared or misdiagnosed cases

Bo Huang etal.,2018[22]	Malaria	investigate temporal changes in genetic diversity of Plasmodium falciparum and MOI	PCR amplification and statistical analysis	• There is significant decrease in the malarial burden due to reduction in the transmission.	• Incomplete information for the shift of the alleles	• Limitation of the study was not mentioned
Paul O Ouma et al.,2018[23]	Emergency health care in SSA	we presented a complete geocoded inventory of hospital services in SSA	Spatial analysis	• Inequalities in emergency healthcare services varies within SSA countries.<br>• This study reported the first spatial census of hospital services in SSA.	• It was difficult to assess the dynamic population changes in spatial analysis	• Unable to calculate the frequency of transport services
Carolina V N Coll et al., 2020[24]	IPV against women is major public health issue	Identify population group which are vulnerable to IPV exposure.	Health survey	• Highlighted different types of IPV IN LMICs.<br>• It helps to identify the vulnerable group of women.	• self-reported data	• self-reported data
Koussay Dellagi et al.,2016[25]	arboviral infections	This study assess the serological of risk and check of arboviral infection in the Union of Comoros.	Cross-sectional study	• Birds were the main source of transmission of viruses.<br>• This is the first study to report WNV infection in the Comoros archipelago.	• Study design	• The enrolled participants do not fully represent the whole Comorian population.
Changsheng Deng et al., 2018[26]	Large-scale Artemisinin – piperaquine administration in highly epidemic region	Identify the mutations in the PfK13 Kelch-propeller sequence which is linked the plasmodium falciparium ring stage artemisinin survival.	Survey	• Both AP plus PMQLD (AP+PMQLD) and AP alone regimens were well tolerated, no major adverse events recorded.<br>• There was no drug resistance seen in malarial patients with plasmodium falciparium in Comoros.	• Sustain and extend public health	
Tomi F. Akinyemiju et al. 2014	Cancer prevention	We determined five-modifiable cancer risk factors within and between 18 African countries.	Survey	• Various adherence patterns found.<br>• Public health efforts needed to improve the behavior of targeted population	• Data collection	• Social biased opinions
Loïc Epelboin et al.,2016[27]	Human angiostrongyliasis	We assessed the epidemiological and	Descriptive analysis	• HA found as a life-threatening disease and	• Ideal control group	• PCR diagnostic properties.

		diagnostic parameters of Human angiostrongyliasis in Mayotte.		mainly diagnosed in Childs under 2 years.<br>• Public should be aware of *A. fulica* and don't allow their children to contact the snails.		
Olalekan A Uthman et al.,2010[28]	AIDS literature	This study provides an overview on HIV research in SSA	Data search on PubMed	• There is need to improve the HIV research productivity in Africa.<br>• The previous published articles on PubMed showed skewed.	• Language biased	• incorrect citation of origin for authors.
Reinhard Kaiser et al.,2015[29]	measles vaccination	Evaluate the coverage of measles vaccination in Eastern and Northern Africa	Survey	• This study recommended to improve the documentation of immunization.<br>• This study developed template to ensure the vaccination coverage surveys are accurate, and comparable for programme improvement.	• Included studies were not clarified from the authors.	• This study doesn't evaluate the design and its implementation fully.
Ayodeji Emmanuel Iyanda et al., 2020[30]	Fertility knowledge	This study aims to determine the associationship between lack of knowledge on ovulation, unwanted pregnancies and child bearing young women in the SSA.	Survey	• This study revealed that adolescent women reported poor knowledge on ovulation and unwanted pregnancies.<br>• Young female must educate about fertility and sexuality.	• No information available on health cost.	• Previous data of female who had unwanted or unintentional pregnancy which results in abortion or mischarge are not reported in this study
Philimon N. Gona, et al., 2020[31]	HIV/AIDS mortality burden	This study accounts the HIV/AIDS mortality burden in Southern Africa	Descriptive analysis	• Study reported that there was significant decline in the HIV mortality burden.<br>• Still more efforts needed to eradicate this disease.	• No data available on transmission of HIV/AIDS through traditional risk factors.	• Death rate was only estimated with active disease.
Daouda Sissoko et al.,2010[32]	chikungunya fever	To evaluate the clinical parameters based on the diagnosis of CHIKF	Cross sectional study	• fever and polyarthralgia is precise diagnostic tool for CHIKF.<br>• This provides an evidence	• Less equipped territory	• Interviews were not conducted at the time of disease outbreak.

				to support the syndromic surveillance in the health settings where diagnostic facilities may be limited.		
Claudia Filippone et al.,2016[33]	Emerging Hantaviruses	Screening mammals sera for the identification of hantaviruses by molecular analysis.	Phylogenetic analysis	• This study reported that this hantavirus is a new variant of Thailand virus.<br>• This study proposed for further investigation of hantaviruses in rodent hosts and in Haemorrhagic fever with HFRS.	• Limited number of individual samples	• This study does not report the limitation.
Daouda Sissoko et al.,2008[34]	Chikungunya Virus Infection	Seroprevalence and risk factors for CHIKV infection in Mayotte.	Survey	This study reported that one out people have Chikungunya Virus asymptomatic.<br>• This study recommends the need of better surveillance and prevention program of arbovirus infection at large scale.	• Misclassification based on febrile illness	• Impact of socio-economic status
Nicolas Pocquet et al.,2016[35]	Insecticide resistance in disease	This study revealed the mechanism of drug resistance in the disease.	Bioassays	• Low insecticide resistance observed in the main mosquito areas.<br>• IVM strategy is ideal to implement in the Mayotte island. and carefully control vector management.	• Ae. albopictus was major vector of arboviruses	• This study does not report the limitation.

References

1. Kassim SA, Alolga RN, Kassim SM, Assanhou AG, Hongchao L, Aixia M. Socioeconomic Disparities in Health Outcomes and Access to Health Care across Three Islands in Comoros. *Journal of health care for the poor and underserved.* 2017;28(3):1116-1140.
2. Donno D, Mellano MG, Hassani S, et al. Assessing Nutritional Traits and Phytochemical Composition of Artisan Jams Produced in Comoros Islands: Using Indigenous Fruits with High Health-Impact as an Example of Biodiversity Integration and Food Security in Rural Development. *Molecules (Basel, Switzerland).* Oct 20 2018;23(10).
3. Oyekale AS, Maselwa TC. Maternal Education, Fertility, and Child Survival in Comoros. *International journal of environmental research and public health.* Dec 10 2018;15(12).
4. Akombi BJ, Agho KE, Merom D, Renzaho AM, Hall JJ. Child malnutrition in sub-Saharan Africa: A meta-analysis of demographic and health surveys (2006-2016). *PloS one.* 2017;12(5):e0177338.
5. Ouledi A, Toyb M, Aubry P, Gaüzere BA. [Heath history and health challenges in the Union of Comoros in 2012]. *Medecine et sante tropicales.* Oct-Dec 2012;22(4):346-354.
6. Ruget AS, Tran A, Waret-Szkuta A, et al. Spatial Multicriteria Evaluation for Mapping the Risk of Occurrence of Peste des Petits Ruminants in Eastern Africa and the Union of the Comoros. *Frontiers in veterinary science.* 2019;6:455.
7. Ortuno-Gutierrez N, Younoussa A, Randrianantoandro A, et al. Protocol, rationale and design of PEOPLE (Post ExpOsure Prophylaxis for LEprosy in the Comoros and Madagascar): a cluster randomized trial on effectiveness of different modalities of implementation of post-exposure prophylaxis of leprosy contacts. *BMC infectious diseases.* Dec 5 2019;19(1):1033.
8. Papa Mze N, Ndiaye YD, Diedhiou CK, et al. RDTs as a source of DNA to study Plasmodium falciparum drug resistance in isolates from Senegal and the Comoros Islands. *Malaria journal.* Sep 29 2015;14:373.
9. de Almeida L, Williams R, Soares DM, Nesbitt H, Wright G, Erskine W. Aflatoxin levels in maize and peanut and blood in women and children: The case of Timor-Leste. *Scientific reports.* Sep 11 2019;9(1):13158.
10. Ben Ali Mbaé S, Mlindassé M, Mihidjaé S, Seyler T. Food-poisoning outbreak and fatality following ingestion of sea turtle meat in the rural community of Ndrondroni, Mohéli Island, Comoros, December 2012. *Toxicon : official journal of the International Society on Toxinology.* Sep 15 2016;120:38-41.
11. Gaüzère BA, Aubry P. [History of human epidemic and endemic diseases in the southwest Indian Ocean]. *Medecine et sante tropicales.* May 1 2013;23(2):145-157.
12. Avanzi C, Lécorché E, Rakotomalala FA, et al. Population Genomics of Mycobacterium leprae Reveals a New Genotype in Madagascar and the Comoros. *Frontiers in microbiology.* 2020;11:711.
13. Salam RA, Syed B, Syed S, et al. Maternal nutrition: how is Eastern and Southern Africa faring and what needs to be done? *African health sciences.* Jun 2015;15(2):532-545.
14. Mokdad AH, Jaber S, Aziz MI, et al. The state of health in the Arab world, 1990-2010: an analysis of the burden of diseases, injuries, and risk factors. *Lancet (London, England).* Jan 25 2014;383(9914):309-320.

15. Dada Y, Milord F, Frost E, et al. The Indian Ocean paradox revisited: HIV and sexually transmitted infections in the Comoros. *International journal of STD & AIDS.* Sep 2007;18(9):596-600.
16. Pagès F, Houze S, Kurtkowiak B, Balleydier E, Chieze F, Filleul L. Status of imported malaria on Réunion Island in 2016. *Malaria journal.* May 24 2018;17(1):210.
17. Elyamine AM, Afzal J. Phenanthrene Mitigates Cadmium Toxicity in Earthworms Eisenia fetida (Epigeic Specie) and Aporrectodea caliginosa (Endogeic Specie) in Soil. Oct 27 2018;15(11).
18. Gebremedhin S. Prevalence and differentials of overweight and obesity in preschool children in Sub-Saharan Africa. *BMJ open.* Dec 23 2015;5(12):e009005.
19. Singh GK, Azuine RE, Siahpush M. Global Inequalities in Cervical Cancer Incidence and Mortality are Linked to Deprivation, Low Socioeconomic Status, and Human Development. *International journal of MCH and AIDS.* 2012;1(1):17-30.
20. AlQarni ZA, Yunus F, Househ MS. Health information sharing on Facebook: An exploratory study on diabetes mellitus. *Journal of infection and public health.* Nov-Dec 2016;9(6):708-712.
21. Camuset G, Lafarge S, Borgherini G, et al. Leprosy on Reunion Island, 2005-2013: Situation and Perspectives. *PLoS neglected tropical diseases.* Apr 2016;10(4):e0004612.
22. Huang B, Tuo F, Liang Y, et al. Temporal changes in genetic diversity of msp-1, msp-2, and msp-3 in Plasmodium falciparum isolates from Grande Comore Island after introduction of ACT. *Malaria journal.* Feb 20 2018;17(1):83.
23. Ouma PO, Maina J, Thuranira PN, et al. Access to emergency hospital care provided by the public sector in sub-Saharan Africa in 2015: a geocoded inventory and spatial analysis. *The Lancet. Global health.* Mar 2018;6(3):e342-e350.
24. Coll CVN, Ewerling F, García-Moreno C, Hellwig F, Barros AJD. Intimate partner violence in 46 low-income and middle-income countries: an appraisal of the most vulnerable groups of women using national health surveys. 2020;5(1):e002208.
25. Dellagi K, Salez N, Maquart M, et al. Serological Evidence of Contrasted Exposure to Arboviral Infections between Islands of the Union of Comoros (Indian Ocean). *PLoS neglected tropical diseases.* Dec 2016;10(12):e0004840.
26. Deng C, Huang B, Wang Q, et al. Large-scale Artemisinin-Piperaquine Mass Drug Administration With or Without Primaquine Dramatically Reduces Malaria in a Highly Endemic Region of Africa. *Clinical infectious diseases : an official publication of the Infectious Diseases Society of America.* Nov 13 2018;67(11):1670-1676.
27. Epelboin L, Blondé R, Chamouine A, et al. Angiostrongylus cantonensis Infection on Mayotte Island, Indian Ocean, 2007-2012. *PLoS neglected tropical diseases.* May 2016;10(5):e0004635.
28. Uthman OA. Pattern and determinants of HIV research productivity in sub-Saharan Africa: bibliometric analysis of 1981 to 2009 PubMed papers. *BMC infectious diseases.* Mar 5 2010;10:47.
29. Kaiser R, Shibeshi ME, Chakauya JM, et al. Surveys of measles vaccination coverage in eastern and southern Africa: a review of quality and methods used. *Bulletin of the World Health Organization.* May 1 2015;93(5):314-319.
30. Iyanda AE, Dinkins BJ, Osayomi T, Adeusi TJ, Lu Y, Oppong JR. Fertility knowledge, contraceptive use and unintentional pregnancy in 29 African countries: a cross-sectional study. *International journal of public health.* May 2020;65(4):445-455.

31. Gona PN, Gona CM, Ballout S, et al. Burden and changes in HIV/AIDS morbidity and mortality in Southern Africa Development Community Countries, 1990-2017. *BMC public health.* Jun 5 2020;20(1):867.
32. Sissoko D, Ezzedine K, Moendandzé A, Giry C, Renault P, Malvy D. Field evaluation of clinical features during chikungunya outbreak in Mayotte, 2005-2006. *Tropical medicine & international health : TM & IH.* May 2010;15(5):600-607.
33. Filippone C, Castel G, Murri S, et al. Discovery of hantavirus circulating among Rattus rattus in French Mayotte island, Indian Ocean. *The Journal of general virology.* May 2016;97(5):1060-1065.
34. Sissoko D, Moendandze A, Malvy D, et al. Seroprevalence and risk factors of chikungunya virus infection in Mayotte, Indian Ocean, 2005-2006: a population-based survey. *PloS one.* Aug 26 2008;3(8):e3066.
35. Pocquet N, Darriet F, Zumbo B, et al. Insecticide resistance in disease vectors from Mayotte: an opportunity for integrated vector management. *Parasites & vectors.* Jul 1 2014;7:299.
